# Prehospital lactate analysis in suspected sepsis improves detection of patients with increased mortality risk: an observational study

**DOI:** 10.1186/s13054-024-05225-2

**Published:** 2025-01-21

**Authors:** Maria Andersson, Karin Fröderberg Schooner, Viktor Karlsson Werther, Thomas Karlsson, Lina De Geer, Daniel B. Wilhelms, Martin Holmbom, Mats Fredrikson, Åse Östholm, Sören Berg, Håkan Hanberger

**Affiliations:** 1 Department of Biomedical and Clinical Sciences, Linköping University, Linköping, Sweden; 2 Department of Infectious Diseases in Region Östergötland, Linköping University Hospital, Linköping, Sweden; 3 Department of Emergency Medicine, Vrinnevi Hospital Norrköping, Norrköping, Sweden; 4 Department of Anaesthesiology and Intensive Care, Linköping University Hospital, Linköping, Sweden; 5 Department of Urology in Östergötland, Linköping University Hospital, Linköping, Sweden; 6 Department of Experimental and Clinical Medicine, Linköping University, Linköping, Sweden; 7 Department of Health, Medicine and Caring Sciences, Linköping University, Linköping, Sweden; 8 Department of Cardiothoracic and Vascular Surgery, Linköping University Hospital, Linköping, Sweden; 9 Department of Emergency Medicine, Local Health Care Services in Central Östergötland, Region Östergötland, Linköping, Sweden

**Keywords:** Lactate, Mortality, Sepsis, Infection, Triage, RETTS, NEWS2, Prehospital, Emergency department, Risk stratification score

## Abstract

**Background:**

Rapid, adequate treatment is crucial to reduce mortality in sepsis. Risk stratification scores used at emergency departments (ED) are limited in detecting all septic patients with increased mortality risk. We assessed whether the addition of prehospital lactate analysis to clinical risk stratification tools improves detection of patients with increased risk for rapid deterioration and death in sepsis.

**Methods:**

A10-month observational study with consecutive, prospective prehospital inclusion of adult patients with suspected sepsis. Prehospital lactate was used as a continuous variable and in intervals. Analyses of patient subgroups with high and lower priorities according to Rapid Emergency Triage and Treatment System (RETTS) and National Early Warning Score 2 (NEWS2) were performed. Primary outcome was 30-day mortality, secondary outcomes were sepsis at the ED and in-hospital mortality.

**Results:**

In all, 714 patients were included with a 30-day mortality of 10%. Among the 322 cases (45%) fulfilling Sepsis-3 criteria, the 30-day mortality was 14%. Prehospital lactate was higher among non-survivors (2.6 vs 2.0 mmol/L, *p* < 0.001). Mortality at different lactate intervals were: 6.7%, at 0–2 mmol/l; 10.0% at > 2–3 mmol/l; 19.2% at > 3–4 mmol/l; and 17.0% at levels > 4 mmol/l. The highest RETTS priority (red) group had higher lactate levels than the lower (non-red) priority group (2.5 vs 1.9 mmol/L, *p* < 0.001). In the non-red group, prehospital lactate was higher among non-survivors (2.4 vs 1.8 mmol/L, *p* = 0.002). In the multivariable regression analysis, prehospital lactate > 3 mmol/l was a predictor of 30-day mortality (OR 2.20, *p* = 0.009) This association was even stronger in the lower priority RETTS non-red group (OR 3.02, *p* = 0.009). Adding prehospital lactate > 3 mmol/l increased identification of non-survivors from 48 to 68% in the RETTS red group and from 77 to 85% for the NEWS2 ≥ 7 group.

**Conclusion:**

The addition of a prehospital lactate level > 3 mmol/l improved early recognition of individuals with increased mortality risk in a cohort with suspected sepsis admitted to the ED. This was particularly evident in patients whose risk stratification scores did not indicate severe illness. We suggest that the addition of prehospital lactate analysis could improve recognition of subjects with suspected sepsis and increased mortality risk.

**Graphical Abstract:**

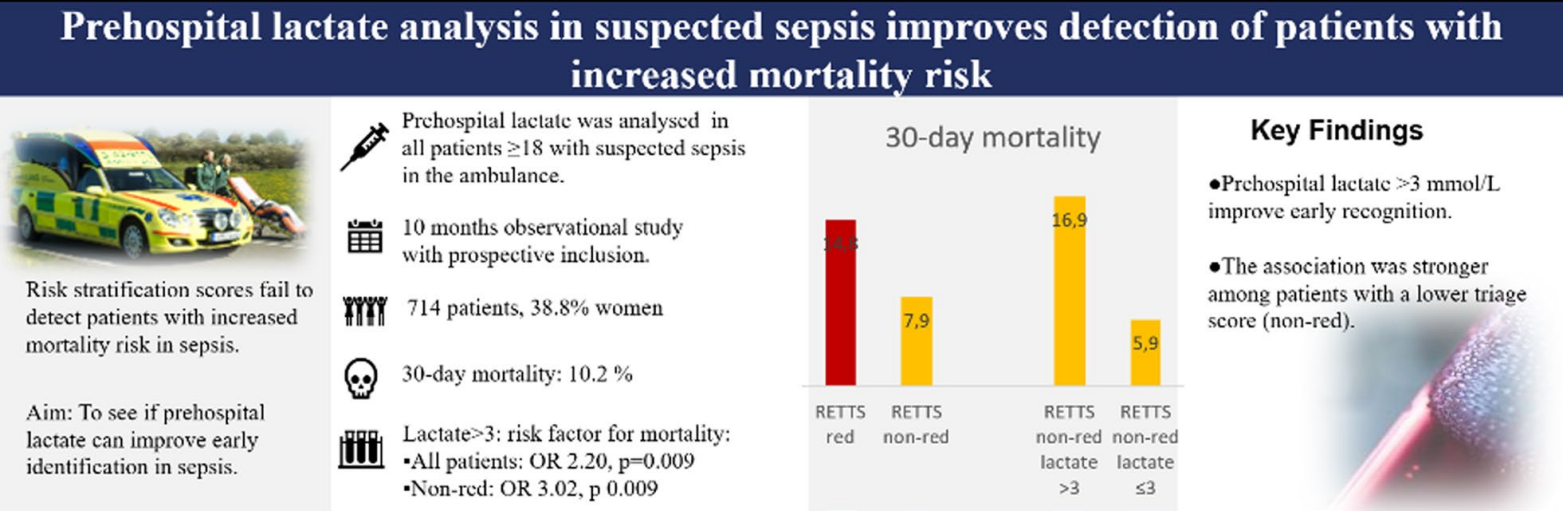

**Supplementary Information:**

The online version contains supplementary material available at 10.1186/s13054-024-05225-2.

## Background

Sepsis is a life-threatening syndrome with organ dysfunction caused by a dysregulated host response to infection [1]. Mortality from sepsis is high, ranging from 10–15% in sepsis and up to 40% in septic shock [1–3]. Time to adequate antibiotic treatment has been identified as an important prognostic factor where shorter time to adequate antibiotic treatment improves survival [4–7]. This is particularly true for patients with septic shock [4, 7]. Early detection of sepsis is essential if we are to achieve timely treatment.

When the patient arrives at the emergency department (ED), it is difficult to foresee the progression of the septic condition and to predict the risk for deterioration. There are various tools for risk stratification that aim to identify patients in need of urgent treatment, but none has adequate sensitivity and specificity. The tools commonly used in Sweden are the Rapid Emergency Triage and Treatment System (RETTS) [8] and the National Early Warning Score 2 [9].

The predictive accuracies of RETTS and NEWS2 have varied. A recent study validating the ability of prehospital RETTS and NEWS2 to predict 30-day mortality in an unselected cohort, showed an area under receiver operating characteristic curve (AUROC) of 0.680 for NEWS2 and 0.666 for RETTS [10]. In an ED study, the ability of RETTS to predict 30-day mortality was 0.758 for all presentations and 0.700 in cases of general infection or fever [11]. Among patients aged ≥ 60 years with suspected sepsis, a NEWS2 ≥ 5 had a low AUROC of 0.56 when predicting mortality [12]. Additionally, older patients often present with vague symptoms, complicating early identification [13].

Increased lactate levels are associated with poor prognosis and mortality in patients with sepsis [14–16], and it has been suggested that serum lactate could be included in risk stratification scores to increase their ability to detect sepsis and predict mortality in patients with infection [17–20]. Prehospital lactate analysis could potentially lead to earlier detection of severe sepsis. In an unselected cohort, elevated prehospital lactate was shown to predict mortality and discriminate patients at risk of death in the group categorized as low risk by NEWS2 [21]. In another unselected prehospital cohort however, lactate measurement did not help in predicting sepsis or time-sensitive conditions [22, 23]. Among sepsis patients, a prehospital lactate > 4 mmol/l was shown to be a notable risk factor for mortality [24], suggesting prehospital lactate to be a possible tool to improve detection of patients with sepsis and increased mortality risk.

The aim of this study was to determine whether adding prehospital lactate to currently used risk stratification tools can improve the detection of patients with increased risk for rapid deterioration and death in sepsis.

## Materials and methods

### Study design and setting

In this prospective observational study, adult patients with suspected sepsis were consecutively included in the ambulance before arrival to the ED. The suspicion of sepsis by the ambulance nurse and paramedic was either through signs and symptoms of infection together with deteriorated vital parameters (defined as RETTS red, RETTS orange or a positive qSOFA) or through concern for sepsis based on clinical experience of the ambulance crew. Lactate was analyzed in the ambulance on all patients.

Inclusion criteria: Patients ≥ 18 years with suspected infection and either a red or orange RETTS score (the two most urgent triage priority levels), a positive qSOFA (quick Sequential Organ Failure Assessment score) [1], or clinical suspicion of sepsis were included in the ambulance.

**Fig. 1:**
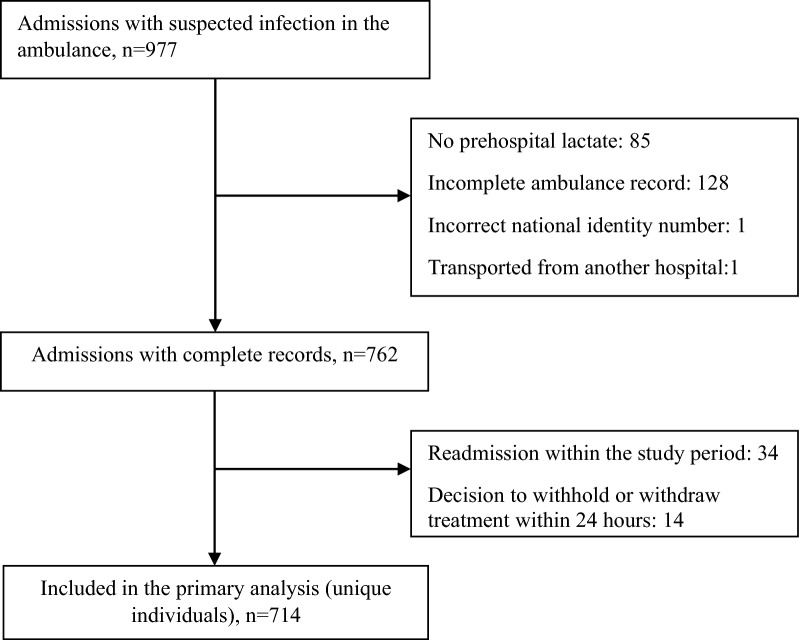
Flowchart for inclusion and exclusion

Exclusion criteria: Patients were excluded from further analysis if information regarding triage or prehospital lactate level was absent, or if they were primarily treated at another hospital. In the case of patients with multiple visits to the ED within the study period only the first visit was included in the analysis. Cases where treatment was withheld or withdrawn within 24 h after admission were also excluded from the analyses (Fig. 1).

Outcome: Primary outcome was 30-day mortality. Secondary outcomes were in-hospital mortality and sepsis on arrival at the ED.

Setting: The study was performed over a 10-month period starting July 18, 2017, in the county of Östergötland, Sweden, with a population of approximately 460,000. The county has a single ambulance organization serving one tertiary care university hospital and two general hospitals. (Additional file 1).

In the ambulance, the crew consisting of one nurse and a paramedic or a second nurse, checked the vital parameters and obtained information on emergency symptoms and signs needed for the triage according to RETTS. Prehospital serum lactate was analyzed in the ambulance using a point-of-care device and reported to the ED. The result of the lactate analysis was available to the physician but there was no specific instruction to act upon the lactate value. Patients included in the study received standard care, that is treatment decided by the ambulance crew and the attending physician in the ED.

## Data collection

A questionnaire to document the inclusion criteria was incorporated in the ambulance medical record form. Reason for prehospital contact, suspicion of infection and sepsis, initial vital parameters, triage status according to RETTS, prehospital lactate level, and treatment with oxygen and intravenous fluids were also recorded prospectively in the ambulance.

Patient data from the ED were collected retrospectively from the medical records. This was due to the high workload in the emergency units making prospective registration difficult, and also since some data were not available until later during and after the hospital stay. This data set included: some vital parameters, laboratory data, treatment in the ED, care time, level of care, mortality, and any care limitation.

## Scores

RETTS is a triage system based on vital signs (blood pressure, heart rate, respiratory rate, oxygen saturation, consciousness, and body temperature) and Emergency Symptoms and Signs (ESS) from the patient’s history. Priority levels are indicated by colors: Red = level 1 – immediately life threatening; Orange = level 2 – emergency, may become life threatening; Yellow = level 3 – urgent but not life threatening; Green = level 4 – semi-urgent, no threat to life; blue = level 5 – no urgency, needs treatment when time permits. The highest priority, determined by any single vital sign or ESS, dictates the triage color [8]. In this study, high priority refers to RETTS red, and lower priorities to the other RETTS levels.

NEWS2 was calculated from the vital parameters recorded in the ambulance. In this study, high priority refers to NEWS2 ≥ 7 and lower priorities to NEWS2 < 7.

Comorbidity according to the Charlson Comorbidity Index (CCI) [25] and diagnoses according to ICD-10 documented within 24 months prior to the date of admission were obtained from the patient-administration system. (Additional file 2).

Sequential Organ Failure Assessment score (SOFA) [26] score was calculated on initial vital parameters documented in the ED. Missing data were assumed to be normal, generating zero SOFA points. If no information on previous organ dysfunction was available, pre-existing organ function was assumed to be normal [27].

Calculating the SOFA score outside the Intensive Care Unit presents difficulties in correctly evaluating the respiratory component. PaO_2_ was estimated from pulse oximetry data according to conversion table used by Aarke [28] and Valik [29]. FiO_2_ was estimated according to Rhee, assuming each 1 L/minute of oxygen flow rate increases FiO_2_ by 0.04 above ambient air FiO_2_ (0.21) [27]. In the absence of respiratory support, the respiratory component of the SOFA score yields a maximum 2 points [26].

## Prehospital lactate analysis

Prehospital lactate analysis was performed on venous blood samples using the Statstrip Lactate Xpress®, from Nova Biomedicals. All ambulances in the region were equipped with this point-of-care device. Before implementation of prehospital lactate analysis, ambulance staff were trained in using the device. Handling and calibration were performed according to the manufacturer’s instructions.

## Definitions

### Sepsis

Sepsis was defined according to the Sepsis-3 criteria as an increase by two points or more in the SOFA score due to infection.

Septic shock was defined as sepsis requiring vasopressor treatment to reach a mean arterial pressure (MAP) of 65 mmHg despite adequate resuscitation and in combination with a serum lactate ≥ 2 mmol/l [1].

### Infection

Infection was based on documented signs and symptoms of infection, laboratory tests (white blood cell count, C-reactive protein, and procalcitonin) and microbiological analyses on admission, and on medical records stating the diagnosis and treatment of infection during admission. Any ambiguity was resolved through discussion with the infectious disease specialists in the team.

## Limitations of level of care

Data on withholding intensive care, cardiopulmonary resuscitation (CPR), ventilator treatment, dialysis, or withdrawal of all active treatment within 24 h of admission, were registered.

## Data validation

Data reliability was validated by repeat classification of 10% of all patients regarding NEWS2, SOFA-score, and prehospital lactate value by the first author (MA). In case of disagreement, this was settled through discussion within the research team.

## Statistical analysis

Data were analyzed using SPSS version 29. Normally distributed data are presented as mean (SD) and non-parametric variables as median (IQR).

Normally distributed, quantitative variables were compared using Student´s t-test and categorical variables using the chi^2^ test or Fishers exact test as appropriate. For non-parametric data, Mann–Whitney U-test was used. Univariate analyses were performed on the primary outcome 30-day mortality and the secondary outcome sepsis on arrival at the ED.

Two-sided P values were used and a *p* value < 0.05 was considered statistically significant. No imputation was needed as few data were missing.

Prehospital lactate was analyzed both as a continuous variable and as interval groups (0–2, > 2–3, > 3–4, > 4 mmol/l). Logistic regression analysis, with lactate as a continuous variable and in interval groups was performed to identify a cut-off level predicting 30-day mortality. Statistically significant differences between survivors and non-survivors in the univariate analyses were included, as was gender. Subgroup analyses on highest (red) and remaining (non-red) triage priorities according to RETTS, were performed. Logistic regressions using the same variables were made on the secondary outcomes. The analyses were checked for overfitting by stepwise exclusion of variables, removing the variable with the highest *p*-value, until we reached a maximum of one variable per 10 of the least common outcome.

Univariate analyses were also made on prehospital lactate in different age-groups.

## Results

In all, 714 patients were included in the analyses (Fig. 1), 437 men (61.2%) and 277 women (38.8%). Mean age was 75.5 years, 30-day mortality was 10.2%, and in-hospital mortality was 6.6%. The number of patients fulfilling Sepsis-3 criteria was 322 (45.1%). Of the remaining patients, 281 (39.4%) had infections without fulfilling the Sepsis-3 criteria and 111 (15.5%) had other diagnoses than infection. Among the patients with other diagnoses than infection the most common were: neurological 22%, surgical 21%, cardiovascular 14% and pulmonary diseases 10%. No significant difference in mortality was observed between men and women (10.5 vs 9.7%) (Table 1).

**Table 1:** Patient characteristics and demographics

	Total *n* = 714	Survivors *n* = 641	Non-survivors *n* = 73	P value	No sepsis *n* = 392	Sepsis *n* = 322	P value
Age (years) (SD)	75.5 (14.2)	74.8 (14.9)	81.7 (9.1)	< *0.001*	74.1 (16.3)	77.3 (11.9)	*0.003*
Female sex (%)	277 (38.8)	250 (39)	27 (37)	0.738	163 (41.6)	114 (35.4)	0.092
In hospital mortality (%)	47 (6.6)	–	–		18 (4.6)	29 (9.0)	*0.018*
30-day mortality (%)	73 (10.2)	–	–		29 (7.4)	44 (13.7)	*0.006*
90-day mortality (%)	109 (15.3)	–	–		53 (13.5)	56 (17.4)	0.152
*Pre-existing comorbidity (%)*							
Myocardial infarction	106 (14.8)	93 (14.5)	13 (17.8)	0.452	56 (14.3)	50 (15.5)	0.642
Congestive heart failure	167 (23.4)	144 (22.5)	23 (31.5)	0.084	86 (21.9)	81 (25.2)	0.312
Peripheral vascular disease	63 (8.8)	54 (8.4)	9 (12.3)	0.265	30 (7.7)	33 (10.2)	0.224
Cerebrovascular disease	120 (16.8)	106 (16.5)	14 (19.2)	0.567	67 (17.1)	53 (16.5)	0.822
Dementia	60 (8.4)	44 (6.9)	16 (21.9)	< *0.001*	31 (7.9)	29 (9.0)	0.599
Chronic pulmonary disease	152 (21.3)	134 (20.9)	18 (24.7)	0.458	83 (21.2)	69 (21.4)	0.934
Connective tissue or rheumatic disease	72 (10.1)	65 (10.1)	7 (9.6)	0.882	29 (7.4)	43 (13.4)	*0.009*
Peptic ulcer disease	16 (2.2)	14 (2.2)	2 (2.7)	0.674	8 (2.0)	8 (2.5)	0.690
Mild liver disease	21 (2.9)	20 (3.1)	1 (1.4)	0.713	10 (2.6)	11 (3.4)	0.496
Diabetes without chronic complications	134 (18.8)	119 (18.6)	15 (20.5)	0.681	73 (18.6)	61 (18.9)	0.913
Diabetes with chronic complications	89 (12.5)	82 (12.8)	7 (9.6)	0.432	52 (13.3)	37 (11.5)	0.475
Paraplegia or hemiplegia	29 (4.1)	25 (3.9)	4 (5.5)	0.526	20 (5.1)	9 (2.8)	0.120
Renal disease	101 (14.1)	87 (13.6)	14 (19.2)	0.193	53 (13.5)	48 (14.9)	0.597
Cancer, not metastatic	107 (15.0)	91 (14.2)	16 (21.9)	0.080	63 (16.1)	44 (13.7)	0.370
Moderate or severe liver disease	7 (1.0)	5 (0.8)	2 (2.7)	0.155	1 (0.3)	6 (1.9)	0.050
Metastatic carcinoma	36 (5.0)	29 (4.5)	7 (9.6)	0.083	18 (4.6)	18 (5.6)	0.544
HIV/AIDS	0	0	0		0	0	
Charlson Comorbidity Score (weighted updated) (Median, IQR)	2.0 (3.0)	2.0 (3.0)	3.0 (2.0)	< *0.001*	2.0 (3.0)	2.0 (3.0)	0.198
Any limitation of level of care on admission (%)	67 (9.4)	44 (6.9)	23 (31.5)	< *0.001*	24 (6.1)	43 (13.4)	< *0.001*
Any limitation of level of care within 24 h after admission (%)	112 (15.7)	75 (11.7)	37 (50.7)	< *0.001*	45 (11.5)	67 (20.8)	< *0.001*

Data are presented as no. (%), mean (SD) or median (IQR). Pearson χ2, Fisher’s exact test, t-test or Mann–Whitney U, are used as appropriate.* P* values < 0.05 are shown in italics

**Fig. 2:**
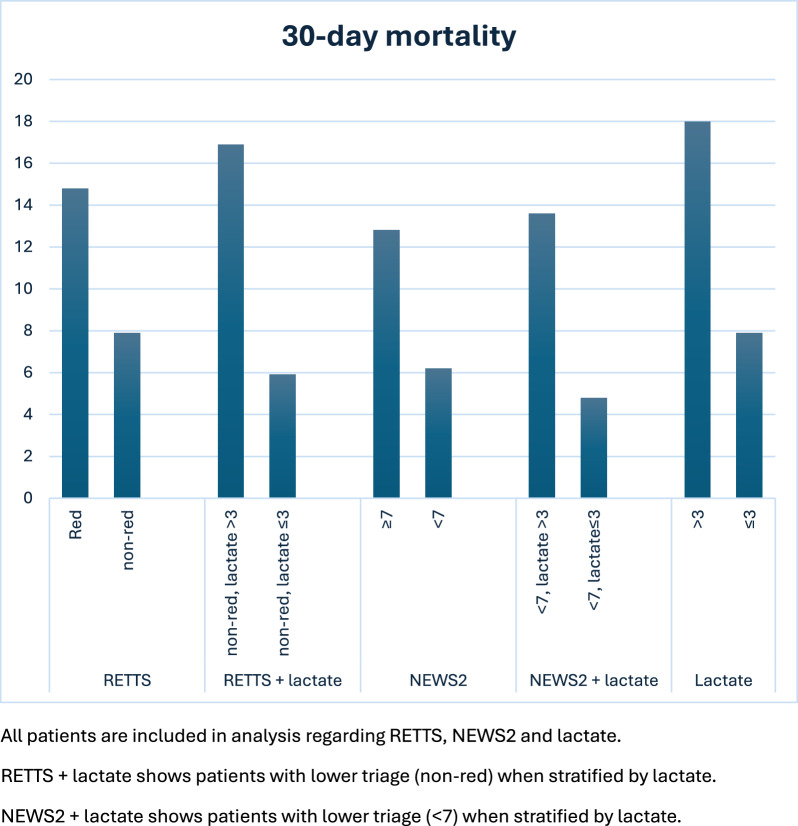
Mortality according to different risk stratification groups

Of the 14 patients excluded due to withdrawal of active treatment within 24 h, one survived and 13 died. Patients with the higher risk stratification scores were older: RETTS red vs non-red (77.2 vs 74.8 years, *p* = 0.047); and NEWS2 ≥ 7 vs < 7 (77.6 vs 74.1 years, *p* < 0.05). Patients triaged higher also and had higher lactate levels: RETTS red vs non-red 2.5 (2.2) vs 1.9 (1.4) mmol/l, p < 0.001; and NEWS2 ≥ 7 vs < 7 2.2 (1.9) vs 1.9 mmol/l (1.4), *p* < 0.004 (median and IQR). There was no difference in comorbidity according to CCI between patients with higher and lower risk stratification scores. Thirty-day mortality in the subgroups were: RETTS red 14.8%, RETTS non-red 7.9%; NEWS 2 ≥ 7 12.8%; and NEWS 2 < 7 6.2% (Table 2, Fig. 2).

**Table 2:** Prehospital symptoms of infection, triage, prehospital lactate, and treatment

	Total *n* = 714	Survivors *n* = 641	Non-survivors *n* = 73	P value	No sepsis *n* = 392	Sepsis *n* = 322	P value
*Symptoms of infection (%)*							
Fever/chills *n* = 709	596 (84.1)	546 (85.8) *n* = 636	50 (68.5)	< *0.001*	315 (81.2) *n* = 388	281 (87.5) *n* = 321	*0.021*
Decline in general condition *n* = 708	313 (44.2)	280 (44.1) *n* = 635	33 (45.2)	0.856	156 (40.3) *n* = 387	157 (48.9) *n* = 321	*0.022*
Rapid deterioration *n* = 708	491 (69.4)	437 (68.7) *n* = 636	54 (75.0)	0.273	253 (65.0) *n* = 389	238 (74.6) *n* = 319	*0.006*
Confusion *n* = 708	218 (30.8)	193 (30.4) *n* = 635	25 (34.2)	0.499	105 (27.1) *n* = 387	113 (35.2) *n* = 321	*0.021*
Gastrointestinal *n* = 707	239 (33.8)	210 (33.1) *n* = 635	29 (40.3) *n* = 72	0.221	124 (32.0) *n* = 388	115 (36.1) *n* = 319	0.252
Respiratory symptoms, *n* = 709	396 (55.9)	342 (53.8) *n* = 363	54 (74.0)	< *0.001*	187 (48.1) *n* = 389	209 (65.3) *n* = 320	< *0.001*
Urinary tract *n* = 703	218 (31.0)	198 (31.4) *n* = 631	20 (27.8) *n* = 72	0.531	116 (30.0) *n* = 387	102 (32.3) *n* = 316	0.511
Headache, upper airways, ears *n* = 704	107 (15.2)	102 (16.1) *n* = 632	5 (6.9) *n* = 72	*0.039*	66 (17.1) *n* = 386	41 (12.9) *n* = 318	0.122
Skin, wound, vascular catheter *n* = 706	91 (12.9)	85 (13.4) *n* = 633	6 (8.2) *n* = 73	0.209	55 (14.2) *n* = 387	36 (11.3)	0.248
*Risk factors for infection (%)*							
Recent surgery, trauma, childbirth *n* = 708	69 (9.7)	62 (9.8) *n* = 635	7 (9.6)	0.962	40 (10.3) *n* = 388	29 (9.1) *n* = 320	0.578
Immunosupression *n* = 706	131 (18.6)	116 (18.3) *n* = 633	15 (20.5)	0.644	69 (17.8) *n* = 388	62 (19.5) *n* = 318	0.560
*Risk stratification*							
RETTS triage (%) *n* = 700				*0.016*			< *0.001*
-red	229 (32.7)	195 (31.0) *n* = 629	34 (47.9) *n* = 71		99 (26.1) *n* = 380	130 (40.6) *n* = 320	
-orange	442 (63.1)	407 (64.7)	35 (49.3)		259 (68.2)	183 (57.2)	
-other	29 (4.1)	27 (4.3)	2 (2.8)		22 (5.8)	7 (2.2)	
NEWS2 (SD)	7.5 (3.2)	7.4 (3.2)	8.9 (3.0)	< *0.001*	6.5 (2.9)	8.8 (3.1)	< *0.001*
NEWS 2 ≥ 7p (%)	439 (61.5)	383 (59.8)	56 (76.7)	*0.005*	189 (48.2)	250 (77.6)	< *0.001*
NEWS 2 ≥ 5p (%)	580 (81.2)	512 (79.9)	68 (93.2)	*0.006*	283 (72.2)	297 (92.2)	< *0.001*
Suspected sepsis *n* = 696	535 (76.9)	476 (76.0) *n* = 626	59 (84.3) *n* = 70	0.121	273 (71.7) *n* = 381	262 (83.2) *n* = 315	< *0.001*
*Prehospital lactate*							
Prehospital lactate mmol/l (median, IQR)	2.1 (1.6)	2.0 (1.6)	2.6 (2.0)	< *0.001*	1.9 (1.4)	2.3 (1.8)	< *0.001*
Prehospital lactate, intervals (%)				*0.001*			< *0.001*
-0–2	357 (50.0)	333 (52.0)	24 (32.9)		223 (56.9)	134 (41.6)	
- > 2–3	190 (26.0)	171 (26.7)	19 (26.0)		94 (24.0)	96 (29.8)	
- > 3–4	73 (10.2)	59 (9.2)	14 (19.2)		37 (9.4)	36 (11.2)	
- > 4	94 (13.2)	78 (12.2)	16 (21.9)		38 (9.7)	56 (17.4)	
Prehospital lactate > 3 (%)	181 (25.4)	137 (21.4)	30 (41.1)	< *0.001*	75 (19.1)	92 (28.6)	*0.003*
*Triage* + *prehospital lactate (%)*							
Prehospital lactate > 3 mmol/l or RETTS red *n* = 700	312 (44.6)	264 (42.0) *n* = 629	48 (67.6) *n* = 71	< *0.001*	144 (37.9) *n* = 380	168 (52.5) *n* = 320	< *0.001*
Prehospital lactate > 3 mmol/l or NEWS2 ≥ 7	483 (67.6)	421 (65.7)	62 (84.9)	< *0.001*	217 (55.4)	266 (82.6)	< *0.001*
Sepsis-3 criteria positive (%)	322 (45.1)	278 (43.4)	44 (60.3)	*0.006*	0	322	
Other diagnosis than infection (%)	111 (15.5)	104 (16.2)	7 (9.6)	0.138	111 (28.3)	0	< *0.001*
*Prehospital treatment (%)*							
Oxygen	451 (63.2)	389 (60.7)	62 (84.9)	< *0.001*	194 (49.5)	257 (79.8)	< *0.001*
Intravenous fluid	500 (70.0)	452 (70.5)	48 (65.8)	0.400	253 (64.5)	247 (76.7)	< *0.001*
First level of care (%)				*0.011*			< *0.001*
-Discharged from ED	82 (11.5)	80 (12.5)	2 (2.7)		66 (16.8)	16 (5.0)	
-General ward	539 (75.5)	483 (75.4)	56 (76.7)		301(76.8)	238 (73.9)	
-Intermediate or intensive care	93 (13.0)	78 (12.2)	15 (20.5)		25 (6.4)	68 (21.1)	

Data are presented as no. (%), mean (SD) or median (IQR). Pearson χ2, Fisher’s exact test, t-test or Mann–Whitney U, are used as appropriate. P values < 0.05 are shown in italics

Data validation by repeat classification of 10% of all patients regarding NEWS2, SOFA-score, and prehospital lactate revealed a proportion of disagreement of 4.5%.

## Survivor’s vs non-survivors

Non-survivors were older (81.7 vs 74.8 years), had a higher comorbidity burden according to CCI, and had level of care limited almost five times as often as survivors (Table 1).

Non-survivors had a higher triage level according to RETTS and a higher NEWS2, but this failed to detect non-survivors in 52.1% (RETTS red) and 23.3% (NEWS2 ≥ 7) of cases. Prehospital lactate was significantly higher in non-survivors than survivors (2.6 mmol/l vs 2.0 mmol/l, *p* < 0.001). Lactate levels were higher in the RETTS red group than in the non-red group (2.5 vs 1.9 mmol/l, *p* < 0.001), and non-red group non-survivors had a significantly higher lactate compared to survivors (2.4 vs 1.8 mmol/l, *p* = 0.002). In the whole study group, prehospital lactate > 2 mmol was significantly associated with mortality (13.7% vs 6.7%, *p* 0.002). Mortality rates for the different lactate integer intervals were: 0–2 mmol/l 6.7%; > 2–3 mmol/l 10.0%; > 3–4 mmol/l 19.2%; and for lactate > 4 mmol/l 17.0%. Thus, the mortality was nearly doubled at lactate > 3 mmol/l. Corresponding figures for RETTS non-red were: 0–2 mmol/l 5.2%; > 2–3 mmol/l 7.4%; > 3–4 mmol/l 14.0%; and for lactate > 4 mmol/l 20.0% (Additional file 3). Since the mortality was distinctly increased at > 3 mmol/l, this level was pragmatically chosen for further statistical evaluation.

Prehospital lactate > 3 mmol/l was more common among non-survivors than survivors (41.1% vs 21.4%, *p* < 0.001) and identified 38% (14/37) of non-survivors in the RETTS non-red group (Table 3). An increased triage priority for all patients with a prehospital lactate > 3 mmol would yield a number needed to treat (NNT) of 9.1, to identify patients with suspected sepsis and increased mortality risk, compared to a NNT of 16,4 with a cutoff > 2 mmol/l.

**Table 3:** Prehospital lactate and 30-day mortality in the RETTS red and RETTS non-red groups

	RETTS red *n* = 229			RETTS non-red *n* = 471			p
Prehospital lactate mmol/l, median (IQR)	2.5 (2.2)			1.9 (1.4)			< *0.001*
	Survivors *n* = 195	Non-survivors *n* = 34	p	Survivors *n* = 434	Non-survivors *n* = 37	p	
Prehospital lactate mmol/l, median (IQR)	2.4 (2.1)	2.8 (2.1)	0.304	1.8 (1.3)	2.4 (2.1)	*0.002*	
Lactate 0–3 mmol/l (%)	131 (67.2)	19 (55.9)	0.201	365 (84.1)	23 (62.2)	< *0.001*	
Lactate > 3 mmol/l (%)	64 (32.8)	15 (44.1)		69 (15.9)	14 (37.8)		

Data are presented as no. (%), and median (IQR). Pearson χ2 and Mann–Whitney U, are used as appropriate. P values < 0.05 are shown in italics

Inclusion of prehospital lactate > 3 mmol/l in risk stratification assessment increased identification of non-survivors from 48 to 68% together with RETTS red and from 77 to 85% together with NEWS2 ≥ 7 (Table 2). The 30-day mortality among RETTS non-red patients but with a prehospital lactate > 3 mmol/l was 16.9% compared to 14.8% in the RETTS red group. Corresponding figures were 13.6% for NEWS 2 < 7 compared to 12.8% for NEWS2 ≥ 7 (Fig. 2). AUROC for RETTS, NEWS2, lactate, together with sensitivity, specificity, positive and negative predictive value (PPV and NPV) and positive and negative likelihood ratio (LR + and LR-) for different lactate intervals are provided in the Additional files 4 and 5.

Vital parameters in the ambulance and ED are presented in supplementary files (Additional file 6). Notable among presenting symptoms was that fever/chills and upper airway symptoms were more common among survivors and lower respiratory symptoms among non-survivors.

## Sepsis vs no sepsis

Sepsis-3 criteria were met in 43.4% of the survivors and 60.3% of the non-survivors (Table 2).

Patients meeting the Sepsis-3 criteria were older (77.3 vs 74.1 years), had a higher 30-day mortality (13.7 vs 7.4%), and more frequently had level of care limited. There was no significant difference in CCI, but connective tissue or rheumatic disease was more common among patients with sepsis (Table 1).

RETTS and NEWS2 were higher among septic than non-septic patients, but still failed to detect sepsis in 59% (RETTS red) and 22% (NEWS2 ≥ 7). Prehospital lactate was higher among sepsis patients (2.3 mmol/l vs 1.9 mmol/l, *p* < 0.001). Although less than one third of sepsis patients had a prehospital lactate value > 3 mmol/l, this level was significantly more common in this group compared to non-sepsis patients (29% vs 19%, *p* = 0.003) (Table 2).

Sepsis patients more often presented with lower respiratory symptoms and non-specific signs of deterioration in general condition, confusion, and fever/chills (Table 2).

## Multivariable analysis of factors associated with 30-day mortality

We analyzed prehospital lactate in different value intervals and found the value > 3 mmol/l to be a significant predictor of 30-day mortality (OR 2.200, *p* = 0.009) (Table 4 and Additional file 7a–d). Subgroup analysis of RETTS red and non-red triage revealed that this association was mainly in the non-red priority group (OR 3.023, CI 1.312–6.966, *p* = 0.009) compared to RETTS red (OR 1.419, CI 0.588–3.424, *p* = 0.436). (Additional files 8a-b and 9a-d). The study group was also checked for outliers, and exclusion of lactate values > 10 mmol/l from the multivariable analyses strengthened the association.

**Table 4:** Risk factors for 30-day mortality

	Univariable analysis			Multivariable analysis		
	OR	95% CI	p	OR	95% CI	*p*
Female	0.918	0.556–1.515	0.738	0.807	0.454–1.435	0.466
Age	1.049	1.023–1.075	< *0.001*	1.028	0.999–1.057	0.062
Limitation of level of care	7.756	4.620–13.023	< *0.001*	4.471	2.444–8.179	< *0.001*
CCI, update	1.244	1.121–1.380	< *0.001*	1.208	1.062–1.374	*0.004*
Fever/chills	0.358	0.208–0.616	< *0.001*	0.457	0.234–0.894	*0.022*
Respiratory symptoms	2.443	1.416–4.216	*0.001*	1.666	0.882–3.146	0.116
Headache, upper airway, ear symptoms	0.388	0.153–0.986	*0.047*	0.746	0.266–2.094	0.578
Oxygen in the ambulance	3.651	1.886–7.068	< *0.001*	2.737	1.190–6.296	*0.018*
NEWS 2 score	1.159	1.074–1.251	< *0.001*	0.987	0.882–1.104	0.819
RETTS: yellow or green	1*			1*		
RETTS: orange	2.354	0.535–10.359	0.258	0.617	0.119–3.186	0.564
RETTS: red	1.161	0.265–5.086	0.843	0.631	0.130–3.072	0.569
Prehospital lactate > 3 mmol/l	2.567	1.552–4.245	< *0.001*	2.200	1.217–3.977	*0.009*
Sepsis (Sepsis 3 criteria)	1.981	1.209–3.247	*0.007*	1.506 lePara>	0.824–2.752	0.183
Care level: Home from ED	1*			1*		
Care level: General ward	4.638	1.110–19.382	*0.036*	1.662	0.371–7.449	0.507
Care level: Intermediate or intensive care	7.692	1.703–34.752	*0.008*	1.498	0.286–7.862	0.633

Multivariable logistic regression with 30-day mortality as dependent variable and prehospital lactate in two intervals. Included cases 688 (618 survivors and 70 non-survivors), missing cases 26

^*^Reference

P values < 0.05 are shown in italics

Of note is that neither the RETTS nor the NEWS2 was a significant predictor of 30-day mortality in the multivariable analysis. As there was a correlation between RETTS, NEWS2, prehospital lactate level, and sepsis, analyses were also performed including only one of these four at a time in the multivariable regression. Prehospital lactate level remained a significant predictor of 30-day mortality while the other covariates showed no significant association. Furthermore, higher comorbidity burden, limitation of level of care taken within 24 h after arrival and need of oxygen in the ambulance were associated with mortality, whereas fever and chills were associated with survival.

Because limitations of level of care may influence the relationship between several other variables and mortality, the multivariable regression was also performed with this variable excluded, without changing the result for lactate (Additional file 10).

The main analysis (Table 4) and the subgroup analysis of RETTS non-red (Additional file 8b) were checked for overfitting, without any changes in the significance of prehospital lactate > 3 mmol/l as a predictor of 30-day mortality (Additional file 11 a and b).

Multivariable analysis was also made with in-hospital mortality as outcome variable. This showed higher in-hospital mortality if prehospital lactate was > 3 mmol/l (OR 3.141, *p* = 0.001) (Additional file 12a–c).

## Prehospital lactate in the elderly

Subgroup analysis of different age intervals showed a significant difference in prehospital lactate between non-survivors and survivors among patients > 80 years (2.9 vs 1.9 mmol/l, *p* < 0.001). In the unadjusted logistic regression, prehospital lactate > 3 mmol/l was a predictor of 30-day mortality for patients 65–80 years (OR 2.821, *p* = 0.013) and > 80 years (OR 3.150, *p* = 0.001). (Additional file 13a–b).

## Discussion

We found that prehospital lactate analysis improves early identification of patients with higher risk of death in a cohort of patients with suspected sepsis coming to the ED by ambulance. A lactate level > 3 mmol/L was associated with increased mortality in the cohort as a whole, with this association being stronger in the subgroup of patients with a lower triage score.

The two risk stratification tools investigated performed poorly in predicting mortality among patients with suspected sepsis. Addition of prehospital lactate > 3 mmol/l increased the ability to identify patients with a higher mortality risk, especially amongst those with lower risk stratification priority. This finding underscores a critical gap in current risk stratification methods, particularly given the widespread reliance on these tools for early decision-making in emergency settings. The poor performance of these tools highlights the need for integrating additional information, such as lactate to improve the accuracy of mortality predictions. That prehospital lactate is a strong predictor of mortality in patients with lower triage priority is an important finding, suggesting that incorporating lactate measurement into the risk stratification could identify patients in need of urgent treatment that otherwise might pass unrecognized.

The lower triage priority group comprises patients with widely varying degrees of illness severity. Despite the lower median prehospital lactate value in this group, the difference between survivors and non-survivors was more pronounced. This observation is in accordance with findings from previous research on a general ED population that also demonstrated the potential of prehospital lactate in predicting mortality more accurately among patients with low risk according to NEWS2 [21]. Lower risk stratification scores often result in delayed treatment, potentially compromising the quality of care provided. This implies that prehospital screening for high lactate levels could reduce mortality in sepsis by earlier detection and timely treatment.

Even though we use both RETTS and NEWS2 under the epithet "risk stratification scores", they are two different tools developed for different purposes. RETTS is a triage tool and was developed to predict urgency in the ED and not primarily mortality, which could be part of the explanation for its weak association with mortality. NEWS2, on the other hand, is an early warning score, developed to predict severity and mortality on in-hospital patients. Both tools are sparsely validated in the prehospital setting [10, 30].

## Lactate and risk stratification scores

The incidence and mortality of sepsis is high [27, 31]. This increases demands on both triage sensitivity, to identify patients in need of urgent treatment, and specificity, to avoid displacement effects in the ED. Sepsis-3 is supposed to define a more homogenous group of patients [1]. Even so there is a wide variation in mortality and not all patients with sepsis according to Sepsis-3 require urgent treatment. It is notable that no association was observed between Sepsis-3 and 30-day mortality in the adjusted analysis. Early warning scores have been shown to have poor to moderate predictive value for mortality in sepsis [32, 33]. The present study concurs with this, revealing that 52% of patients who died within 30 days were not identified as RETTS red, and amongst those with NEWS 2 ≥ 7 24% would have been missed. In Sweden this is important since a national patient-specific clinical treatment algorithm for sepsis based on RETTS-red or NEWS2 ≥ 7 is currently being introduced [34].

Lactate and risk stratification tools alone do not have sensitivity and specificity enough to identify all infected patients in need of urgent treatment. However, the integration of lactate analysis with current scoring systems could improve detection. Combined scores, such as L(lactate)-qSOFA [17, 18] and L-NEWS2 [19] have been suggested. The L-qSOFA score with a cut-off level of 2 mmol/l has been suggested to increase sensitivity regarding mortality or adverse outcome [17, 18]. The threshold of ≥ 2 mmol/l was also shown to increase sensitivity in sepsis detection in a recent meta-analysis [20], but the low specificity is a problem with its use in the ED setting [18]. In our study, a lower lactate threshold (≥ 2 mmol/l) was associated with increased mortality in the univariate analysis but in the multivariable logistic regression a level > 3 mmol/l was a stronger predictor of mortality. An increased triage priority for all patients with a lactate > 2 mmol would yield a NNT of 16,4, compared to 9,1 with the cutoff at lactate > 3 mmol/l. Obviously, the cutoff of > 3 mmol/l excludes some patients with an increased mortality risk, but using > 2 mmol/l as threshold would increase the number of patients with the highest priority. This could possibly lead to over-triaging a number of patients, causing less efficient medical care. Since point-of-care lactate analysis is now readily available, this should contribute valuable information despite suboptimal sensitivity and specificity.

Prehospital analysis increases early recognition of patients with a high mortality risk and enables early therapeutic intervention. Early detection of sepsis in the ambulance has been shown to predict mortality in critical ill patients [35] and to improve prediction of mortality among patients with low risk according to NEWS2 [21]. A prehospital lactate > 3 mmol/l could be used pragmatically to identify patients with suspected sepsis and high mortality risk (Fig. 2).

Even though prehospital lactate analysis can improve detection, not all patients who die within 30 days are detected. With timely sepsis treatment, some deaths could be avoided, but other deaths may be more related to premorbid comorbidity or acquired morbidity due to sepsis. In this study, the lower in-hospital mortality and the fact that mortality was associated with higher age and degree of comorbidity supports the latter. A higher long-term mortality risk associated with sepsis was also shown in a recent study [36].

## Early detection among the elderly

RETTS and NEWS2 are the most common risk stratification scores used in Sweden. They are, as the SOFA score, based on the ability of the patient to produce symptoms. However, symptoms may be disguised, particularly in the elderly, by medication, a less responsive immune system, or by chronic conditions that mask symptoms. This results in non-specific complaints that often lead to a lower triage priority despite a thrice higher mortality (33 vs 11%) compared to patients with sepsis and more specific symptoms [13]. In the present study, patients with higher triage priority were older, but neither triage priority nor age was found to be predictive of mortality in the multivariable analysis. Rather CCI and limitation of level of care were associated with outcome, indicating higher risk among frail individuals with comorbidity.

Increased mortality has previously been shown among patients 65 years and older with infection and a lactate level of > 2 mmol/l [37, 38] Sanguanwit et al. showed NEWS2 to have low predictive ability regarding mortality and sepsis among the elderly, but a significant difference in lactate levels between survivors and non-survivors [12]. Dundar showed a low to moderate increase in accuracy when combining NEWS2 with lactate (NEWS-L) [39]. In our study, a prehospital lactate > 3 mmol/L was seen to be predictive of mortality risk in sepsis, with OR increasing with higher age. These findings indicate that lactate may be helpful in improving early identification of elderly patients with sepsis and increased mortality risk.

## Sepsis or mortality as outcome

Sepsis according to Sepsis-3 results in an in-hospital mortality greater than 10% [1], which is in line with the 9% in-hospital mortality among sepsis patients in this study. However, 30-day mortality among patients with suspected sepsis not fulfilling Sepsis-3 criteria was 7.4% in our study. The outcome variables sepsis and mortality identify different groups. Sepsis includes some patients without high risk for deterioration and death, while excluding some patients with infection and high mortality risk. All-cause mortality, on the other hand, includes patients with diagnoses other than sepsis as the cause of death. As the purpose of early identification is to reduce avoidable mortality and morbidity, we chose 30-day mortality as our main outcome variable.

The optimal time interval for measuring mortality caused by sepsis is also a key consideration. In a study by Durantez-Fernandez, the ability of NEWS2, lactate level, and the combined measure NEWS2-L to predict 2-day, 7-day, 14-day, and 30-day mortality was examined, revealing higher predictive accuracy in the shorter time interval [19]. This is in line with our results, showing a significant predictive ability of lactate regarding 30-day mortality (OR 2.200, p 0.009) and even stronger prediction of the secondary outcome, in-hospital mortality (OR 3.141, p 0.001). All-cause mortality risk remains even after discharge following sepsis, augmented by both pre-existing and acquired comorbidity [36].

## Strengths and weaknesses

This is an observational study with prospective inclusion but with retrospective data, and thus has the limitations of a retrospective study. The availability of results from prehospital lactate measurements in the ED is a confounding factor in that treatment could have been adapted according to the lactate level. In the study, there was no specific instruction to act upon the lactate value, yet the result was known to the clinician and probably influenced treatment and thereby probably primary mortality outcome. However, such bias should reduce the predictive value of lactate since mortality would likely be reduced by more aggressive management.

This study only included patients with suspected sepsis and not all patients with infection, and thus conclusions can only be drawn regarding patients with suspected sepsis arriving at the ED by ambulance. Approximately 25% of the patients were excluded due to incomplete ambulance records or missing prehospital lactate. It is possible that this was not at random, potentially resulting in selection bias.

This study included some patients that did not fulfil the criteria for infection, and other patients who did not need hospital admission. It is uncertain if this reduced the predictive value of lactate.

Triage tools are not primarily designed to predict mortality or severity, but urgency, and to prioritize the patient flow. Triage is based on mortality risk but also on other factors. Using a triage system that is largely based on factors other than mortality risk, for example workload and demand for resources, the patient's triage level can be affected by these external factors. Therefore, the triage does not provide a reliable measure of severity and mortality, which could lead to misinterpretations. Nevertheless, RETTS, which is the most common triage tool in Sweden, has been validated against mortality and need of hospital admission [8, 40].

Generalization of our findings is limited by the use of the RETTS triage tool as this is not widespread internationally. Although NEWS2 was calculated for all patients to enhance external validity, the inclusion criteria were not based on NEWS2, thus limiting the ability to draw definitive conclusions on its accuracy.

Although our study was conducted in three hospitals, including one tertiary care and two general hospitals, the results should be validated in additional cohorts.

## Conclusions

Among patients with suspected sepsis, the use of prehospital lactate levels to complement two commonly used risk stratification scores improved identification of patients with increased mortality risk. This was especially valid in patients not recognized as being seriously ill due to a lower triage score. We suggest using prehospital lactate analysis to improve triage algorithms in patients with suspected sepsis, where levels > 3 mmol/l predict increased mortality risk.

## Supplementary Information

Supplementary Material 1: Additional file 1–13 (590 KB)

## Abbreviations

AUROC: Area under receiver operating characteristic curve
CCI: Charlson Comorbidity Index
CPR: Cardiopulmonary resuscitation
ED: Emergency department
ESS: Emergency Symptoms and Signs
FiO_2_: Fraction inspired oxygen
LR-: Negative likelihood ratio
LR +: Positive likelihood ratio
MAP: Mean arterial pressure
NEWS2: National Early Warning Score 2
NNT: Number needed to treat
NPV: Negative predictive value
OR: Odds ratio
PaO_2_: Partial pressure of oxygen
PPV: Positive predictive value
RETTS: Rapid Emergency Triage and Treatment System
SOFA score: Sequential Organ Failure Assessment score
qSOFA: Quick Sequential Organ Failure Assessment score
