# Supplementary material for: Prehospital lactate analysis in suspected sepsis improves detection of patients with increased mortality risk: an observational study
Source: Crit Care. 2025 Jan 21;29:38. doi: 10.1186/s13054-024-05225-2 (PMC11753079; doi:10.1186/s13054-024-05225-2)
Supplement: Supplementary file 1 — Supplementary Material 1: Additional file 1–13 (590 KB) [file 13054_2024_5225_MOESM1_ESM.pdf]

Additional file 1. Health care organisation in Region Östergötland, a county of South East Sweden, 2017-2018

Prehospital organisation	One regional prehospital organization responsible for guidelines, quality, and coordination.<br>Two providers of ambulance transports.<br>Approximately 15 ambulances<br>Each ambulance was equipped for prehospital lactate analysis (see material and methods).
Tertiary Care University Hospital	302 beds<br>Non-cardiothoracic mixed general intensive care unit with 9 beds<br>Intermediate care unit with 5 beds, with possibility to vasopressor treatment and non-invasive ventilation.<br>ED with approximately 49000 visits/year.
General hospital 1	Approximately 220 beds<br>General intensive care unit with 6 beds<br>Intermediate care unit with one bed, with possibility to vasopressor treatment and non-invasive ventilation.<br>ED with approximately 49000 visits/year.
General hospital 2	56 beds.<br>Intermediate care unit with 4 beds, with possibility to vasopressor treatment and non-invasive ventilation.<br>ED with approximately 25000 visits/year.

Additional file 2. Coding Algorithms for Defining Comorbidities in ICD-9-CM and ICD-10 Administrative Data, used for Charlson Comorbidity Index.

Comorbid Condition	ICD-9 Diagnosis Codes	ICD-10 Diagnosis Codes	Updated Weight (Quaan 2011)
Myocardial Infarction	410, 412	I21, I22, I25.2	0
Congestive Heart Failure	398.91, 402.01, 402.11, 402.91, 404.01, 404.03, 404.11, 404.13, 404.91, 404.93, 425.4–425.9, 428 (hosp),<br>398, 402, 425, 428 (med)	I09.9, I11.0, I13.0, I13.2, I25.5, I42.0, I42.5–I42.9, I43, I50, P29.0	2
Peripheral Vascular disease	093.0, 437.3, 440, 441, 443.1–443.9, 447.1, 557.1, 557.9, V43.3 (hosp)<br>440, 441, 443, 447, 557 (med)	I70, I71, I73.1, I73.8, I73.9, I77.1, I79.0,<br>I79.2, K55.1, K55.8, K55.9, Z95.8, Z95.9	0
Cerebrovascular Disease	362.34, 430–438 (hosp)<br>430–438 (med)	G45, G46, H34.0, I60–I69	0
Dementia	290, 294.1, 331.2 (hosp)<br>290, 294, 331 (med)	F00–F03, F05.1, G30, G31.1	2

Chronic Pulmonary Disease	416.8, 416.9, 490–505, 506.4, 508.1, 508.8 (hosp)<br>416, 490–496, 500–505 (med)	I27.8, I27.9, J40–J47, J60–J67 J68.4, J70.1, J70.3	1
Connective Tissue Disease-<br>Rheumatic Disease	446.5, 710.0–710.4, 714.0–714.2, 714.8, 725 (hosp)<br>446, 710, 714, 725 (med)	M05, M06, M31.5, M32–M34, M35.1, M35.3, M36.0	1
Peptic Ulcer Disease	531–534	K25–K28	0
Mild Liver Disease	070.22, 070.23, 070.32, 070.33, 070.44,<br>070.54, 070.6, 070.9, 570, 571, 573.3,<br>573.4, 573.8, 573.9, V42.7 (hosp)<br>070, 570, 571, 573 (med)	B18, K70.0–K70.3, K70.9, K71.3–K71.5,<br>K71.7, K73, K74, K76.0, K76.2–K76.4,<br>K76.8, K76.9, Z94.4	2
Diabetes without Chronic Complications	250.0–250.3, 250.8, 250.9 (hosp)<br>250 (med)	E10.0, E10.1, E10.6, E10.8, E10.9, E11.0, E11.1, E11.6, E11.8, E11.9, E12.0, E12.1, E12.6, E12.8, E12.9, E13.0, E13.1, E13.6, E13.8, E13.9, E14.0, E14.1, E14.6, E14.8, E14.9	0
Diabetes with Chronic Complications	250.4–250.7<br>(med n/a)	E10.2–E10.5, E10.7, E11.2–E11.5, E11.7, E12.2–E12.5, E12.7, E13.2–E13.5, E13.7, E14.2–E14.5, E14.7	1
Paraplegia and Hemiplegia	334.1, 342, 343, 344.0–344.6, 344.9 (hosp),<br>334, 342–344 (med)	G04.1, G11.4, G80.1, G80.2, G81, G82, G83.0–G83.4, G83.9	2
Renal Disease	403.01, 403.11, 403.91, 404.02, 404.03, 404.12, 404.13, 404.92, 404.93, 582, 583.0–583.7, 585, 586, 588.0. V42.0, V45.1, V56 (hosp),<br>403, 582, 583, 585,	I12.0, I13.1, N03.2–N03.7, N052–N05.7, N18, N19, N25.0, Z49.0–Z49.2, Z94.0, Z99.2	1
Cancer *	140–172, 174–195.8, 200–208, 238.6 (hosp),<br>140–172, 174–195, 200–208, 238 (med)	C00–C26, C30–C34, C37–C41, C43, C45–C58, C60–C76, C81–C85, C88, C90–C97	2
Moderate or Severe Liver<br>Disease	456.0–456.2, 572.2–572.4, 572.8 (hosp), 456, 572 (med)	I85.0, I85.9, I86.4, I98.2, K70.4, K71.1, K72.1, K72.9, K76.5–K76.7	4
Metastatic Carcinoma**	196–199	C77–C80	6
HIV/AIDS	042–044	B20–B22, B24	4

Abbreviations: AIDS, acquired immunodeficiency syndrome; HIV, human immunodeficiency virus. * Including lymphoma and leukemia, except malignant neoplasm of skin. ** or Metastatic solid tumor.

Additional file 3. 30-day mortality at different lactate intervals.

	Lactate interval	0-2 mmol/l	>2-3 mmol/l	>3-4 mmol/l	>4 mmol/l
All patients	Survivors (%)	333 (93.3)	171 (90.0)	59 (80.8)	78 (83.0)
	Non-survivors (%)	24 (6.7)	19 (10.0)	14 (19.2)	16 (17.0)
RETTS non-red	Survivors (%)	253 (94.8)	112 (92.6)	37 (86)	32 (80)
	Non-survivors (%)	14 (5.2)	9 (7.4)	6 (14.0)	8 (20.0)

Additional file 4. ROC-curves for RETTS, NEWS2 and prehospital lactate

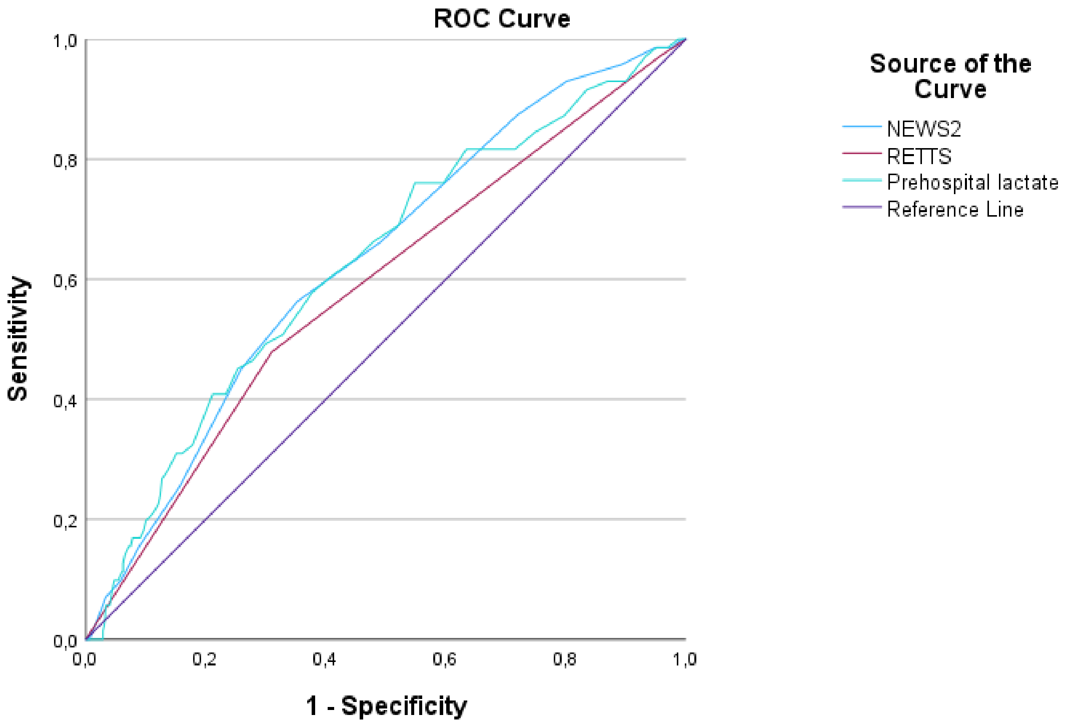

AUC: NEWS2 0.630, RETTS 0.586, prehospital lactate 0.627

Additional file 5. Sensitivity, specificity, PPV, NPV, LR+ and LR- for prehospital lactate at different cutoffs.

	All patients			RETTS non-red			Patients with Sepsis-3		
Lactate	>2	>3	>4	>2	>3	>4	>2	>3	>4
Sensitivity	0.67	0.41	0.21	0.62	0.38	0.22	0.63	0.40	0.25
Specificity	0.51	0.79	0.88	0.58	0.84	0.93	0.42	0.73	0.84
PPV	0.13	0.18	0.17	0.11	0.17	0.20	0.15	0.20	0.20
NPV	0.93	0.91	0.90	0.95	0.94	0.93	0.88	0.89	0.88
LR+	1.367	1.952	1.750	1.476	2.375	3.143	1.086	1.481	1.563
LR-	0.647	0.747	0.898	0.655	0.738	0.839	0.881	0.821	0.893

Additional file 6. Vital parameters in the ambulance and in the ED

	**Survivors n=641**	**Non-survivors n=73**	**P value**	**No sepsis n=392**	**Sepsis n=322**	**P value**
**Vital parameters in the ambulance**						
Body temperature (C)	38.6 (1.1) n=639	38.0 (1.3) n=72	*<0.001*	38.4 (1.2) n=391	38.8 (1.1) n=320	*<0.001*
Systolic blood pressure	133 (28) n=635	121 (28)	<0.001	135 (27) n=390	129 (29) n=318	*0.007*
Diastolic blood pressure	78 (17) n=539	73 (17) n=539	*0.014*	79 (16) n=322	76 (19) n=278	0.054
Mean arterial blood pressure	97 (19) n=539	89 (19) n=61	*0.003*	98 (18) n=322	94 (20) n=278	*0.011*
Heart rate	102 (21) n=640	101 (21) n=71	0.698	99 (20) n=391	105 (21) n=320	*<0.001*
Respiratory rate	27 (8) n=638	30 (8) n=73	*<0.001*	25 (7) n=390	29 (9) n=321	*<0.001*
Saturation (SpO2%), median (IQR)	92 (7)	88 (11)	*<0.001*	94 (6)	90 (7)	*<0.001*
Supplementary Oxygen (%)	21 (3.3) n=637	6 (8.3) n=72	*0.047*	9 (2.3) n=382	18 (5.7) n=318	*0.020*
Supplementary Oxygen l/min, median (IQR)	0 (0)	0 (0)	*0.023*	0 (0)	0 (0)	*0.033*
Reaction level scale, median (IQR)	1 (0)	1 (0)	*0.024*	1 (0)	1 (0)	*<0.001*
**Vital parameters in the ED**						
Body temperature (C)	38.1 (1.0) n=466	37.6 (1.0) n=45	*0.004*	37.9 (1.0) n=265	38.1 (1.1) n=246	*0.004*
Systolic blood pressure (mmHg)	128 (25) n=576	118 (27) n=66	*0.002*	132 (24) n=332	122 (25) n=310	*<0.001*
Diastolic blood pressure (mmHg)	74 (16) n=574	71 (18) n=66	*0.135*	77 (15) n=331	71 (16) n=309	*<0.001*
Mean arterial blood pressure (mmHg)	92 (17) n=574	87 (19) n=66	*0.015*	96 (16) n=331	88 (17) n=309	*<0.001*
Heart rate	97 (20) n=547	98 (22) n=66	0.715	95 (21) n=319	98 (21) n=287	0.065
Respiratory rate	23 (7) n=516	25 (7) n=58	*0.049*	22 (6) n=300	25 (7) n=274	*<0.001*
Saturation (SpO2%), median (IQR)	96 (4)	95 (6)	0.091	97 (4)	95 (5)	*<0.001*
Supplementary Oxygen (%)	281 (49.0) n=573	40 (62.5) n=64	*0.041*	114 (34.5) n=330	207 (67.4) n=307	*<0.001*
Supplementary Oxygen (l/min), median (IQR)	0 (3)	2 (4.8)	*0.014*	0 (2)	3 (4)	*<0-001*
Reaction level scale, median (IQR)	1 (0)	1 (1)	*<0.001*	1 (0)	1 (0)	*<0.001*

Data is presented as no. (%) or mean (SD), if not indicated others. Pearson $\chi 2$, Fisher's exact test or t-test, are used as appropriate. Non-parametric variables are analysed with Mann-Whitney Test and presented as median (IQR). P values <0.05 are shown in italics.

Additional file 7. Analyses on all patients. Logistic regression with 30-day mortality as outcome. Included cases 688 (618 survivors and 70 non-survivors), missing cases 26.

Additional file 7a. Prehospital lactate analysed as a continuous variable.

All patients	Univariate analysis				Multivariable analyses			
	B	p	OR	95% CI	B	p	OR	95% CI
Female	-0.86	0.738	0.918	0.556-1.515	-0.250	0.391	0.779	0.439-1.380
Age	0.047	<0.001	1.049	1.023-1.075	0.029	0.043	1.030	1.001-1.059
Limitation of level of care	2.049	<0.001	7.756	4.620-13.023	1.494	<0.001	4.454	2.448-8.103
CCI, update	0.218	<0.001	1.244	1.121-1.380	0.179	0.006	1.196	1.052-1.360
Fever/chills	-1.026	<0.001	0.358	0.208-0.616	-0.808	0.018	0.446	0.228-0.872
Respiratory symptoms	0.893	0.001	2.443	1.416-4.216	0.509	0.115	1.663	0.884-3.131

Headache, upper airway, ear symptoms	-0.947	0.047	.0388	0.153-0.986	-0.293	0.575	0.746	0.268-2.075
Oxygen in the ambulance	1.295	<0.001	3.651	1.886-7.068	0.976	0.021	2.654	1.161-6.063
NEWS 2 score	0.148	<0.001	1.159	1.074-1.251	-0.014	0.801	0.986	0.881-1.103
RETTS: yellow or green			1				1	
RETTS: orange	0.149	0.843	1.161	0.265-5.086	-0.391	0.628	0.676	0.139-3.297
RETTS: red	0.856	0.258	2.354	0.535-10.359	-0.391	0.640	0.676	0.131-3.483
Prehospital lactate mmol/l	0.148	0.012	1.159	1.033-1.300	0.112	0.131	1.119	0.967-1.295
Sepsis (Sepsis 3 criteria)	0.684	0.007	1.981	1.209-3.247	0.405	0.187	1.499	0.822-2.734
Care level: Home from ED			1				1	
Care level: General ward	1.534	0.036	4.638	1.110-19.382	0.595	0.435	1.813	0.407-8.074
Care level: Intermediate or intensive care	2.040	0.008	7.692	1.703-34.752	0.530	0.530	1.700	0.325-8.883

Additional file 7b. Prehospital lactate in four intervals.

All patients	Univariate analysis				Multivariable analyses			
	B	p	OR	95% CI	B	p	OR	95% CI
Female	-0.86	0.738	0.918	0.556-1.515	-0.204	0.489	0.816	0.458-1.453
Age	0.047	<0.001	1.049	1.023-1.075	0.027	0.063	1.028	0.999-1.058
Limitation of level of care	2.049	<0.001	7.756	4.620-13.023	1.498	<0.001	4.471	2.443-8.182
CCI, update	0.218	<0.001	1.244	1.121-1.380	0.188	0.004	1.207	1.062-1.373
Fever/chills	-1.026	<0.001	0.358	0.208-0.616	-0.777	0.023	0.460	0.235-0.899
Respiratory symptoms	0.893	0.001	2.443	1.416-4.216	0.522	0.110	1.685	0.889-3.194
Headache, upper airway, ear symptoms	-0.947	0.047	.0388	0.153-0.986	-0.276	0.602	0.759	0.269-2.138
Oxygen in the ambulance	1.295	<0.001	3.651	1.886-7.068	1.018	0.017	2.768	1.202-6.375
NEWS 2 score	0.148	<0.001	1.159	1.074-1.251	-0.012	0.838	0.988	0.882-1.107
RETTS: yellow or green			1				1	
RETTS: orange	0.149	0.843	1.161	0.265-5.086	-0.416	0.608	0.659	0.134-3.243
RETTS: red	0.856	0.258	2.354	0.535-10.359	-0.458	0.586	0.632	0.122-3.286
Prehospital lactate 0-2 mmol/l			1				1	
Prehospital lactate 2-3 mmol/l	0.433	0.178	1.542	0.822-2.893	0.249	0.495	1.283	0.627-2.625
Prehospital lactate 3-4 mmol/l	1.192	0.001	3.292	1.611-6.730	0.934	0.027	2.546	1.112-5.830
Prehospital lactate >4 mmol/l	1.046	0.003	2.846	1.443-5.612	0.858	0.036	2.358	1.058-5.255
Sepsis (Sepsis 3 criteria)	0.684	0.007	1.981	1.209-3.247	0.399	0.195	1.491	0.815-2.726
Care level: Home from ED			1				1	
Care level: General ward	1.534	0.036	4.638	1.110-19.382	0.484	0.528	1.622	0.361-7.286
Care level: Intermediate or intensive care	2.040	0.008	7.692	1.703-34.752	0.377	0.656	1.458	0.278-7.652

Additional file 7c. Prehospital lactate in three intervals.

All patients	Univariate analysis				Multivariable analyses			
	B	p	OR	95% CI	B	p	OR	95% CI
Female	-0.86	0.738	0.918	0.556-1.515	-0.208	0.479	0.812	0.457-1.445
Age	0.047	<0.001	1.049	1.023-1.075	0.027	0.062	1.028	0.999-1.058
Limitation of level of care	2.049	<0.001	7.756	4.620-13.023	1.498	<0.001	4.475	2.445-8.188
CCI, update	0.218	<0.001	1.244	1.121-1.380	0.188	0.004	1.207	1.062-1.373

Fever/chills	-1.026	<0.001	0.358	0.208-0.616	-0.775	0.024	0.461	0.235-0.901
Respiratory symptoms	0.893	0.001	2.443	1.416-4.216	0.526	0.105	1.693	0.895-3.201
Headache, upper airway, ear symptoms	-0.947	0.047	.0388	0.153-0.986	-0.283	0.591	0.753	0.268-2.116
Oxygen in the ambulance	1.295	<0.001	3.651	1.886-7.068	1.017	0.017	2.764	1.201-6.362
NEWS 2 score	0.148	<0.001	1.159	1.074-1.251	-0.013	0.821	0.987	0.882-1.105
RETTS: yellow or green			1				1	
RETTS: orange	0.149	0.843	1.161	0.265-5.086	0.412	0.612	0.662	0.135-3.253
RETTS: red	0.856	0.258	2.354	0.535-10.359	-0.457	0.587	0.633	0.122-3.288
Prehospital lactate 0-2 mmol/l			1				1	
Prehospital lactate 2-3 mmol/l	0.433	0.178	1.542	0.822-2.893	0.250	0.493	1.284	0.628-2.627
Prehospital lactate >3 mmol/l	1.111	<0.001	3.038	1.714-5.386	0.894	0.009	2.444	1.250-4.780
Sepsis (Sepsis 3 criteria)	0.684	0.007	1.981	1.209-3.247	0.398	0.196	1.489	0.815-2.723
Care level: Home from ED			1				1	
Care level: General ward	1.534	0.036	4.638	1.110-19.382	0.489	0.523	1.631	0.364-7.316
Care level: Intermediate or intensive care	2.040	0.008	7.692	1.703-34.752	0.376	0.657	1.457	0.277-7.652

Additional file 7d. Prehospital lactate in two intervals.

All patients	Univariate analysis				Multivariable analyses			
	B	p	OR	95% CI	B	p	OR	95% CI
Female	-0.86	0.738	0.918	0.556-1.515	-0.214	0.466	0.807	0.454-1.435
Age	0.047	<0.001	1.049	1.023-1.075	0.027	0.062	1.028	0.999-1.057
Limitation of level of care	2.049	<0.001	7.756	4.620-13.023	1.498	<0.001	4.471	2.444-8.179
CCI, update	0.218	<0.001	1.244	1.121-1.380	0.189	0.004	1.208	1.062-1.374
Fever/chills	-1.026	<0.001	0.358	0.208-0.616	-0.782	0.022	0.457	0.234-0.894
Respiratory symptoms	0.893	0.001	2.443	1.416-4.216	0.510	0.116	1.666	0.882-3.146
Headache, upper airway, ear symptoms	-0.947	0.047	.0388	0.153-0.986	-0.293	0.578	0.746	0.266-2.094
Oxygen in the ambulance	1.295	<0.001	3.651	1.886-7.068	1.007	0.018	2.737	1.190-6.296
NEWS 2 score	0.148	<0.001	1.159	1.074-1.251	-0.013	0.819	0.987	0.882-1.104
RETTS: yellow or green			1				1	
RETTS: orange	0.149	0.843	1.161	0.265-5.086	-0.460	0.569	0.631	0.130-3.072
RETTS: red	0.856	0.258	2.354	0.535-10.359	-0.483	0.564	0.617	0.119-3.186
Prehospital lactate >3 mmol/l	0.943	<0.001	2.567	1.552-4.245	0.788	0.009	2.200	1.217-3.977
Sepsis (Sepsis 3 criteria)	0.684	0.007	1.981	1.209-3.247	0.410	0.183	1.506	0.824-2.752
Care level: Home from ED			1				1	
Care level: General ward	1.534	0.036	4.638	1.110-19.382	0.508	0.507	1.662	0.371-7.449
Care level: Intermediate or intensive care	2.040	0.008	7.692	1.703-34.752	0.404	0.633	1.498	0.286-7.862

Additional file 8a. Logistic regression with 30-day mortality as outcome, with RETTS red. Included cases 223 (190 survivors and 33 non-survivors), excluded cases 6.

All patients	Univariate analysis				Multivariable analyses			
	B	p	OR	95% CI	B	p	OR	95% CI
Female	0.299	0.426	1.349	0.646-2.818	0.227	0.614	1.255	0.520-3.030
Age	0.032	0.074	1.032	0.997-1.068	0.015	0.457	1.016	0.975-1.058
Limitation of level of care	1.842	<0.001	6.310	2.913-13.666	1.611	<0.001	5.008	2.030-12.359
CCI, update	0.073	0.447	1.076	0.891-1.298	0.100	0.404	1.105	0.874-1.397
Fever/chills	-0.711	0.075	0.491	0.224-1.075	-0.858	0.098	0.424	0.154-1.170
Respiratory symptoms	1.113	0.045	3.043	1.025-9.039	1.226	0.049	3.408	1.004-11.570
Headache, upper airway, ear symptoms	-1.579	0.128	0.206	0.027-1.577	-0.756	0.490	0.470	0.055-4.005
Oxygen in the ambulance	1.539	0.040	4.662	1.075-20.226	1.130	0.172	3.096	0.612-15.667
NEWS 2 score	0.110	0.074	1.117	0.990-1.260	0.050	0.573	1.051	0.884-1.251
Prehospital lactate >3 mmol/l	0.480	0.204	1.616	0.771-3.387	0.350	0.436	1.419	0.588-3.424
Sepsis (Sepsis 3 criteria)	0.243	0.525	1.275	0.604-2.691	0.197	0.688	1.217	0.467-3.175
Care level: Home from ED			1				1	
Care level: General ward	0.860	0.418	2.364	0.295-18.918	0.216	0.857	1.242	0.118-13.051
Care level: Intermediate or intensive care	0.850	0.439	2.340	0.271-20.173	-0.282	0.828	0.754	0.059-9.594

Additional file 8b. Logistic regression with 30-day mortality as outcome, with RETTS non-red. Included cases 465 (428 survivors and 37 non-survivors), excluded cases 6.

All patients	Univariate analysis				Multivariable analyses			
	B	p	OR	95% CI	B	p	OR	95% CI
Female	-0.601	0.116	0.548	0.259-1.161	-0.749	0.081	0.473	0.204-1.097
Age	0.057	0.002	1.059	1.022-1.097	0.036	0.094	1.037	0.994-1.081
Limitation of level of care	1.961	<0.001	7.105	3.404-14.813	1.314	0.004	3.722	1.539-9.004
CCI, update	0.280	<0.001	1.323	1.157-1.514	0.245	0.004	1.278	1.083-1.507
Fever/chills	-0.916	0.026	0.400	0.178-0.897	-0.829	0.089	0.437	0.168-1.134
Respiratory symptoms	0.646	0.067	1.907	0.956-3.806	0.193	0.642	1.213	0.537-2.737
Headache, upper airway, ear symptoms	-0.556	0.308	0.574	0.197-1.668	0.059	0.926	1.060	0.308-3.651
Oxygen in the ambulance	1.308	0.002	3.697	1.590-8.599	1.082	0.039	2.951	1.058-8.234
NEWS 2 score	0.104	0.095	1.109	0.982-1.253	-0.028	0.731	0.972	0.827-1.143
Prehospital lactate >3 mmol/l	1.169	0.001	3.220	.1579-6.565	1.106	0.009	3.023	1.312-6.966
Sepsis (Sepsis 3 criteria)	0.843	0.016	2.322	1.172-4.603	0.600	0.143	1.822	0.817-4.065
Care level: Home from ED			1				1	
Care level: General ward	1.739	0.090	5.694	0.763-42.468	0.888	0.404	2.431	0.302-19.549
Care level: Intermediate or intensive care	2.420	0.031	11.250	1.256-100.793	1.307	0.283	3.694	0.340-40.069

Additional file 9. Analyses on RETTS non-red. Logistic regression with 30-day mortality as outcome. Included cases 465 (428 survivors and 37 non-survivors), excluded cases 6.

Additional file 9a. Prehospital lactate as a continuous variable

Other triage color than red	Univariate analysis				Multivariable analyses			
	B	p	OR	95% CI	B	p	OR	95% CI
Female	-0.601	0.116	0.548	0.259-1.161	-0.841	0.053	0.431	0.184-1.012
Age	0.057	0.002	1.059	1.022-1.097	0.041	0.058	1.042	0.999-1.087
Limitation of level of care	1.961	<0.001	7.105	3.404-14.831	1.321	0.003	3.746	1.554-9.032
CCI, update	0.280	<0.001	1.323	1.157-1.514	0.246	0.004	1.279	1.084-1.509
Fever/chills	-0.916	0.026	0.400	1.178-0.897	-0.846	0.084	0.429	0.164-1.121
Respiratory symptoms	0.646	0.067	1.907	0.956-3.806	0.213	0.606	1.237	0.551-2.778
Headache, upper airway, ear symptoms	-0.556	0.308	0.574	0.197-1.668	-0.019	0.976	0.981	0.281-3.426
Oxygen in the ambulance	1.308	0.002	3.697	1.590-8.599	1.045	0.043	2.843	1.031-7.838
NEWS 2 score	0.104	0.095	1.109	0.982-1.253	-0.051	0.539	0.950	0.808-1.118
Prehospital lactate mmol/l	0.233	0.009	1.263	1.060-1.504	0.317	0.006	1.372	1.094-1.722
Sepsis (Sepsis 3 criteria)	0.843	0.016	2.322	1.172-4.603	0.666	0.108	1.947	0.864-4.384
Care level: Home from ED			1				1	
Care level: General ward	1.739	0.090	5.694	0.763-42.468	1.037	0.329	2.820	0.352-22.614
Care level: Intermediate or intensive care	2.420	0.031	11.250	1.256-100.793	1.390	0.250	4.016	0.377-42.814

Additional file 9b. Prehospital lactate in four intervals

Other triage color than red	Univariate analysis				Multivariable analyses			
	B	p	OR	95% CI	B	p	OR	95% CI
Female	-0.601	0.116	0.548	0.259-1.161	-0.850	0.055	0.427	0.179-1.018
Age	0.057	0.002	1.059	1.022-1.097	0.037	0.092	1.038	0.994-1.084
Limitation of level of care	1.961	<0.001	7.105	3.404-14.831	1.308	0.004	3.697	1.524-8.971
CCI, update	0.280	<0.001	1.323	1.157-1.514	0.243	0.004	1.274	1.082-1.501
Fever/chills	-0.916	0.026	0.400	1.178-0.897	-0.802	0.104	0.448	0.171-1.179
Respiratory symptoms	0.646	0.067	1.907	0.956-3.806	0.230	0.581	1.258	0.557-2.841
Headache, upper airway, ear symptoms	-0.556	0.308	0.574	0.197-1.668	-0.035	0.957	0.965	0.267-3.485
Oxygen in the ambulance	1.308	0.002	3.697	1.590-8.599	1.116	0.033	3.054	1.092-8.543
NEWS 2 score	0.104	0.095	1.109	0.982-1.253	-0.039	0.644	0.962	0.817-1.133
Prehospital lactate 0-2 mmol/l			1				1	
Prehospital lactate 2-3 mmol/l	0.373	0.399	1.452	0.611-3.454	0.403	0.412	1.496	0.571-3.921
Prehospital lactate 3-4 mmol/l	1.075	0.038	2.931	1.060-8.100	0.875	0.130	2.399	0.773-7.450
Prehospital lactate >4 mmol/l	1.508	0.002	4.518	1.759-11.604	1.749	0.004	5.752	1.768-18.711
Sepsis (Sepsis 3 criteria)	0.843	0.016	2.322	1.172	0.623	0.133	1.864	0.827-4.204
Care level: Home from ED			1				1	
Care level: General ward	1.739	0.090	5.694	0.763-42.468	1.052	0.329	2.862	0.346-23.661
Care level: Intermediate or intensive care	2.420	0.031	11.250	1.256-100.793	1.264	0.303	3.539	0.320-39.141

Additional file 9c. Prehospital lactate in three intervals.

Other triage color than red	Univariate analysis				Multivariable analyses			
	B	p	OR	95% CI	B	p	OR	95% CI
Female	-0.601	0.116	0.548	0.259-1.161	-0.751	0.081	0.472	0.203-1.097
Age	0.057	0.002	1.059	1.022-1.097	0.038	0.080	1.039	0.995-1.085
Limitation of level of care	1.961	<0.001	7.105	3.404-14.831	1.304	0.004	3.682	1.525-8.890
CCI, update	0.280	<0.001	1.323	1.157-1.514	0.240	0.004	1.272	1.079-1.499
Fever/chills	-0.916	0.026	0.400	1.178-0.897	-0.805	0.099	0.447	0.172-1.165
Respiratory symptoms	0.646	0.067	1.907	0.956-3.806	0.205	0.622	1.227	0.544-2.769
Headache, upper airway, ear symptoms	-0.556	0.308	0.574	0.197-1.668	0.051	0.936	1.052	0.303-3.648
Oxygen in the ambulance	1.308	0.002	3.697	1.590-8.599	1.101	0.035	3.006	1.078-8.385
NEWS 2 score	0.104	0.095	1.109	0.982-1.253	-0.031	0.711	0.970	0.825-1.141
Prehospital lactate 0-2 mmol/l			1				1	
Prehospital lactate 2-3 mmol/l	0.373	0.399	1.452	0.611-3.454	0.392	0.424	1.480	0.566-3.867
Prehospital lactate >3 mmol/l	1.299	0.001	3.667	1.669-8.056	1.253	0.008	3.502	1.395-8.792
Sepsis (Sepsis 3 criteria)	0.843	0.016	2.322	1.172	0.602	0.143	1.826	0.817-4.084
Care level: Home from ED			1				1	
Care level: General ward	1.739	0.090	5.694	0.763-42.468	0.918	0.389	2.505	0.310-20.265
Care level: Intermediate or intensive care	2.420	0.031	11.250	1.256-100.793	1.301	0.285	3.671	0.338-39.852

Additional file 9d. Prehospital lactate in two intervals.

Other triage color than red	Univariate analysis				Multivariable analyses			
	B	p	OR	95% CI	B	p	OR	95% CI
Female	-0.601	0.116	0.548	0.259-1.161	-0.749	0.081	0.473	0.204-1.097
Age	0.057	*0.002*	1.059	1.022-1.097	0.036	0.094	1.037	0.994-1.081
Limitation of level of care	1.961	*<0.001*	7.105	3.404-14.831	1.314	0.004	3.722	1.539-9.004
CCI, update	0.280	*<0.001*	1.323	1.157-1.514	0.245	0.004	1.278	1.083-1.507
Fever/chills	-0.916	*0.026*	0.400	1.178-0.897	-0.829	0.089	0.437	0.168-1.134
Respiratory symptoms	0.646	0.067	1.907	0.956-3.806	0.193	0.642	1.213	0.537-2.737
Headache, upper airway, ear symptoms	-0.556	0.308	0.574	0.197-1.668	0.059	0.926	1.060	0.308-3.651
Oxygen in the ambulance	1.308	*0.002*	3.697	1.590-8.599	1.082	0.039	2.951	1.058-8.234
NEWS 2 score	0.104	0.095	1.109	0.982-1.253	-0.028	0.731	0.972	0.827-1.143
Prehospital lactate >3 mmol/l	1.169	*0.001*	3.322	1.579-6.565	1.106	0.009	3.023	1.312-6.966
Sepsis (Sepsis 3 criteria)	0.843	*0.016*	2.322	1.172-4.603	0.600	0.143	1.822	0.817-4.065
Care level: Home from ED			1				1	
Care level: General ward	1.739	0.090	5.694	0.763-42.468	0.888	0.404	2.431	0.302-19.549
Care level: Intermediate or intensive care	2.420	0.031	11.250	1.256-100.793	1.307	0.283	3.694	0.340-40.069

Additional file 10. Analyses on all patients, with Limitations of level of care excluded. Logistic regression with 30-day mortality as outcome. Included cases 688 (618 survivors and 70 non-survivors), missing cases 26.

All patients	Univariate analysis				Multivariable analyses			
	B	p	OR	95% CI	B	p	OR	95% CI
Female	-0.86	0.738	0.918	0.556-1.515	-0.187	0.511	0.830	0.475-1.448
Age	0.047	<0.001	1.049	1.023-1.075	0.045	0.002	1.046	1.017-1.077
CCI, update	0.218	<0.001	1.244	1.121-1.380	0.202	0.001	1.224	1.082-1.384
Fever/chills	-1.026	<0.001	0.358	0.208-0.616	-0.965	0.003	0.381	0.201-0.721
Respiratory symptoms	0.893	0.001	2.443	1.416-4.216	0.545	0.083	1.724	0.931-3.194
Headache, upper airway, ear symptoms	-0.947	0.047	.0388	0.153-0.986	-0.448	0.377	0.639	0.236-1.726
Oxygen in the ambulance	1.295	<0.001	3.651	1.886-7.068	0.949	0.023	2.584	1.141-5.852
NEWS 2 score	0.148	<0.001	1.159	1.074-1.251	0.032	0.562	1.032	0.927-1.150
RETTS: yellow or green			1				1	
RETTS: orange	0.149	0.843	1.161	0.265-5.086	-0.145	0.863	0.865	0.167-4.471
RETTS: red	0.856	0.258	2.354	0.535-10.359	-0.168	0.836	0.845	0.172-4.165
Prehospital lactate >3 mmol/l	0.943	<0.001	2.567	1.552-4.245	0.778	0.008	2.177	1.230-3.852
Sepsis (Sepsis 3 criteria)	0.684	0.007	1.981	1.209-3.247	0.372	0.209	1.451	0.812-2.591
Care level: Home from ED			1				1	
Care level: General ward	1.534	0.036	4.638	1.110-19.382	0.716	0.348	2.046	0.459-9.116
Care level: Intermediate or intensive care	2.040	0.008	7.692	1.703-34.752	0.756	0.368	2.129	0.411-11.023

Additional file 11. Multivariable logistic regression with reduced number of variables to check for overfitting.

Additional file 11a. Analyses on all patients. Logistic regression with 30-day mortality as outcome. Included cases 688 (618 survivors and 70 non-survivors), missing cases 26.

All patients	Univariate analysis				Multivariable analyses			
	B	p	OR	95% CI	B	p	OR	95% CI
Age	0.047	<0.001	1.049	1.023-1.075	0.027	0.055	1.027	0.999-1.055
Limitation of level of care	2.049	<0.001	7.756	4.620-13.023	1.555	<0.001	4.735	2.681-8.364
CCI, update	0.218	<0.001	1.244	1.121-1.380	0.197	0.002	1.218	1.078-1.376
Fever/chills	-1.026	<0.001	0.358	0.208-0.616	-0.772	0.014	0.462	0.250-0.855
Respiratory symptoms	0.893	0.001	2.443	1.416-4.216	0.473	0.125	1.605	0.876-2.939
Oxygen in the ambulance	1.295	<0.001	3.651	1.886-7.068	0.963	0.009	2.619	1.266-5.417
Prehospital lactate >3 mmol/l	0.943	<0.001	2.567	1.552-4.245	0.762	0.008	2.144	1.225-3.750

Additional file 11b. Analyses on RETTS non-red. Logistic regression with 30-day mortality as outcome. Included cases 465 (428 survivors and 37 non-survivors), excluded cases 6.

RETTS non-red	Univariate analysis				Multivariable analyses			
	B	p	OR	95% CI	B	p	OR	95% CI
Limitation of level of care	1.961	<0.001	7.105	3.404-14.831	1.789	<0.001	5.982	2.759-12.968
CCI, update	0.280	<0.001	1.323	1.157-1.514	0.268	<0.001	1.308	1.127-1.517
Prehospital lactate >3 mmol/l	1.169	0.001	3.322	1.579-6.565	1.131	0.004	3.098	1.439-6.671

Additional file 12. Logistic regression with in-hospital mortality as outcome. Prehospital lactate in two intervals.

Additional file 12a. Analyses on all patients. Included cases 688 (644 survivors and 44 non-survivors), missing cases 26.

All patients	Univariate analysis				Multivariable analyses			
	B	p	OR	95% CI	B	p	OR	95% CI
Female	-0.220	0.490	0.803	0.431-1.497	-0.278	0.438	0.757	0.374-1.531
Age	0.036	0.011	1.037	1.009-1.067	0.024	0.183	1.024	0.989-1.060
Limitation of level of care	1.632	<0.001	5.112	2.761-9.466	0.963	0.010	2.620	1.261-5.441
CCI, update	0.173	0.007	1.189	1.049-1.347	0.147	0.062	1.159	0.992-1.353
Fever/chills	-1.101	<0.001	0.333	0.175-0.631	-1.024	0.010	0.359	0.165-0.780
Respiratory symptoms	1.277	<0.001	3.585	1.706-7.533	0.927	0.034	2.528	1.074-5.951
Headache, upper airway, ear symptoms	-0.990	0.103	0.372	0.113-1.220	-0.380	0.562	0.684	0.189-2.471
Oxygen in the ambulance	1.455	0.001	4.283	1.793-10.232	0.837	0.125	2.309	0.792-6.730
NEWS 2 score	0.177	<0.001	1.193	1.088-1.309	0.025	0.716	1.025	0.896-1.173
RETTS: yellow or green			1				1	
RETTS: orange	0.283	0.786	1.327	0.172-10.251	-0.519	0.637	0.595	0.069-5.118
RETTS: red	1.187	0.254	3.278	0.427-25.185	-0.623	0.583	0.536	0.058-4.956
Prehospital lactate >3 mmol/l	1.435	<0.001	4.201	2.301-7.672	1.144	0.001	3.141	1.560-6.323
Sepsis (Sepsis 3 criteria)	0.721	0.020	2.057	1.120-3.776	0.499	0.185	1.647	0.787-3.448
Care level: Home from ED	-19.386	0.997	0.000		-17.553	0.997	0.000	
Care level: General ward	-0.881	0.011	0.414	0.210-0.819	-0.214	0.614	0.807	0.352-1.852
Care level: Intermediate or intensive care			1				1	

Additional file 12b. Analysis on RETTS red. Included cases 223 (200 survivors and 23 non-survivors), missing cases 6.

All patients	Univariate analysis				Multivariable analyses			
	B	p	OR	95% CI	B	p	OR	95% CI
Female	-0.023	0.958	0.977	0.408-2.339	-0.034	0.947	0.966	0.350-2.667
Age	0.030	0.146	1.030	0.990-1.072	0.021	0.388	1.021	0.974-1.070
Limitation of level of care	1.693	<0.001	5.433	2.255-13.093	1.390	0.009	4.013	1.425-11.301
CCI, update	0.089	0.416	1.093	0.882-1.356	0.127	0.342	1.136	0.874-1.476
Fever/chills	-0.780	0.086	0.458	0.188-1.117	-1.067	0.067	0.344	0.110-1.077
Respiratory symptoms	1.475	0.051	4.370	0.996-19.180	1.595	0.050	4.929	1.001-24.265
Headache, upper airway, ear symptoms	-1.145	0.273	0.318	0.041-2.465	-0.302	0.787	0.740	0.083-6.619

Oxygen in the ambulance	1.101	0.146	3.006	0.681-13.278	0.395	0.642	1.484	0.281-7.846
NEWS 2 score	0.129	0.071	1.137	0.989-1.308	0.074	0.456	1.077	0.886-1.308
Prehospital lactate >3 mmol/l	0.723	0.096	2.060	0.879-4.827	0.543	0.282	1.722	0.640-4.634
Sepsis (Sepsis 3 criteria)	0.266	0.550	1.304	0.546-3.117	0.173	0.755	1.189	0.400-3.532
Care level: Home from ED	-19.351	0.999	0.000		-18.094	0.999	0.000	
Care level: General ward	-0.317	0.494	0.729	0.294-1.805	0.058	0.918	1.060	0.352-3.187
Care level: Intermediate or intensive care			1				1	

Additional file 12c. Analyses on RETTS non-red. Included cases 465 (444 survivors and 21 non-survivors), missing cases 6.

All patients	Univariate analysis				Multivariable analyses			
	B	p	OR	95% CI	B	p	OR	95% CI
Female	-0.502	0.309	0.606	0.231-1.590	0.769	0.162	0.463	0.158-1.363
Age	0.041	0.055	1.042	0.999-1.087	0.028	0.307	1.028	0.975-1.085
Limitation of level of care	1.232	*0.015*	3.430	1.270-9.265	0.365	0.551	1.440	0.434-4.783
CCI, update	0.217	*0.012*	1.242	1.048-1.472	0.174	0.103	1.189	0.966-1.465
Fever/chills	-1.109	*0.028*	0.330	0.123-0.888	-1.148	0.053	0.317	0.099-1.014
Respiratory symptoms	1.060	*0.031*	2.886	1.100-7.575	0.615	0.271	1.849	0.618-5.534
Headache, upper airway, ear symptoms	-0.684	0.364	0.504	0.115-2.211	-0.205	0.809	0.815	0.154-4.298
Oxygen in the ambulance	1.614	*0.011*	5.020	1.458-17.284	1.281	0.088	3.601	0.826-15.700
NEWS 2 score	0.134	0.100	1.143	0.975-1.341	0.004	0.972	1.004	0.814-1.238
Prehospital lactate >3 mmol/l	1.754	*<0.001*	5.775	2.365-14.101	1.727	*<0.001*	5.626	2.049-15.444
Sepsis (Sepsis 3 criteria)	1.136	*0.016*	3.114	1.232-7.867	0.809	0.132	2.246	0.783-6.445
Care level: Home from ED	-19.222	0.997	0.000		-17.588	0.997	0.000	
Care level: General ward	-1.064	0.071	0.345	0.109-1.094	-0.486	0.501	0.615	0.149-2.536
Care level: Intermediate or intensive care			1				1	

Additional file 13. Prehospital lactate and 30-day mortality in different age intervals.

Additional file 13a. Lactate as a continuous variable in the logistic regression.

		Univariate analysis			Univariate logistic regression			
Prehospital lactate median (IQR)	n	Survivors n=641	Non-survivors n=73	p	B	p	OR	CI
All patients	714	2.0 (1.6)	2.6 (2.0)	*<0.001*	0.148	*0.012*	1.159	1.033-1.300
18-45	36	1.95 (1.9) n=36						
>45-65	91	2.1 (1.6) n=87	1.8 (0.43) n=4	0.519	-0.438	0.412	0.646	0.227-1.838
>65-80	285	2.1 (1.5) n=258	2.5 (1.8) n=27	0.053	0.132	0.153	1.141	0.952-1.369
>80	302	1.9 (1.5) n=260	2.9 (2.2) n=42	*<0.001*	0.271	*0.004*	1.311	1.093-1.574

Data is presented as mean (SD). T-test and Mann-Whitney U Test are used. P values <0.05 are shown in italics.

Additional file 13b. Lactate at the cutoff >3 mmol/l the unadjusted analysis.

		Univariate analysis			Univariate logistic regression, lactate >3 mmol/l			
Prehospital lactate median (IQR)	n	Survivors n=641	Non-survivors n=73	p	B	p	OR	CI
All patients	714	2.0 (1.6)	2.6 (2.0)	*<0.001*	0.943	*<0.001*	2.567	1.552-4.245
18-45	36	1.95 (1.9) n=36						
>45-65	91	2.1 (1.6) n=87	1.8 (0.43) n=4	0.519	-18.415	0.998	0.000	
>65-80	285	2.1 (1.5) n=258	2.5 (1.8) n=27	0.053	1.037	*0.013*	2.821	1.250-6.367
>80	302	1.9 (1.5) n=260	2.9 (2.2) n=42	*<0.001*	1.147	*0.001*	3.150	1.589-6.246

Data is presented as mean (SD). T-test and Mann-Whitney U Test are used. P values <0.05 are shown in italics.
